# CDCA-Derived NE3TA Conjugate for Liver-Selective ^64^Cu PET Imaging

**DOI:** 10.1021/acsomega.5c13607

**Published:** 2026-04-01

**Authors:** Haixing Wang, Siyuan Ren, Nilantha Bandara, Shuyuan Zhang, Rachael Fujimori, Hong Ha Nguyen, David D. L. Minh, Buck E. Rogers, Hyun-Soon Chong

**Affiliations:** † Department of Chemistry, 2455 Illinois Institute of Technology, Chicago, Illinois 60616-3717, United States; ‡ Department of Radiation Oncology, 7548 Washington University, Saint Louis, Missouri 63110-1010, United States

## Abstract

Positron emission tomography (PET) is a highly sensitive and quantitative imaging modality that has been applied for noninvasive visualization of biochemical and metabolic processes *in vivo*. While PET has the potential to assess hepatobiliary function and transport, liver-selective PET tracers have not been successfully translated into clinical applications. Copper-64 (^64^Cu) is a positron-emitting radionuclide that has been widely explored for PET imaging of bioactive molecules. We developed an effective bifunctional chelator 3p-*C*-NE3TA that can securely and rapidly sequester ^64^Cu under mild conditions. Herein, we report the synthesis and evaluation of a CDCA-derived chelator conjugate, 3p-*C*-NE3TA-chenodeoxycholic acid (CDCA), for potential applications in liver-selective PET imaging. The 3p-*C*-NE3TA-CDCA conjugate was efficiently radiolabeled with ^64^Cu at room temperature. The resulting ^64^Cu-labeled 3p-*C*-NE3TA-CDCA conjugate demonstrated high stability in human serum and prominent and selective hepatic uptake with favorable clearance, as evidenced by very low activity levels in blood and nontarget tissues in healthy mice at 4 h postinjection. Taken together, these results show that ^64^Cu-labeled 3p-*C*-NE3TA-CDCA exhibits excellent radiolabeling efficiency, high *in vitro* and *in vivo* stability, and marked liver-selective biodistribution.

## Graphical abstract

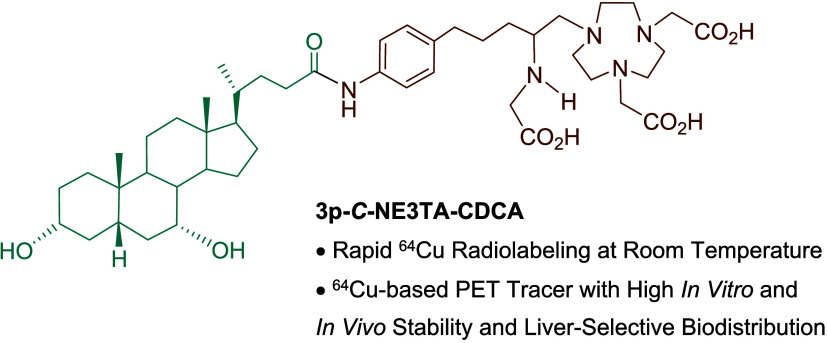

## Introduction

Liver diseases represent a substantial and increasing health burden and remain a major contributor to mortality worldwide.^1,2^ Chronic liver diseases arising from metabolic and cholestatic disorders often progress to advanced stages including hepatic fibrosis and cirrhosis and hepatocellular carcinoma (HCC), which are associated with poor prognosis.^2–5^

Noninvasive and sensitive imaging techniques are therefore needed to enable the functional assessment of hepatobiliary processes. Bile acids are essential regulators of hepatobiliary physiology and govern lipid absorption, enterohepatic circulation, and hepatic secretion.^6,7^ Dysregulation of bile acid homeostasis is implicated in the pathogenesis of diverse liver diseases including primary biliary cholangitis (PBC) and metabolic dysfunction-associated steatotic liver disease (MASLD).^8,9^ Bile acid clearance has been used as a functional marker of hepatobiliary secretion and liver health.^6,7^ Bile acid analogues have been explored as positron emission tomography (PET) tracers for functional imaging of hepatobiliary transport.^10–13^ In particular, ^11^C-labeled bile acid analogue cholylsarcosine ([*N*-methyl-^11^C]­cholylsarcosine, ^11^C–CSar) was shown to enable assessment of hepatobiliary transport kinetics and reported as a promising tool for in vivo evaluation of hepatobiliary function.^10,11^ However, PET tracers based on the short-lived ^11^C (*t*_1/2_ = 20.3 min) have limited utility for mapping hepatic uptake and clearance over extended imaging windows.^14–16^

Copper-64 (^64^Cu) is a positron-emitting radionuclide with a relatively long physical half-life (*t*_1/2_ = 12.7 h) and has been extensively explored for PET imaging of biomarkers, particularly in oncology.^17–23^ We have previously reported a series of bimodal chelators containing both acyclic and macrocyclic binding moieties for the coordination of various radionuclides.^24–30^ The design of the chelators in the series of NE3TA, NETA, NEPA, and DEPA was based on the rationale that the donor atoms in the macrocyclic backbone provide secure binding of metallic radionuclides, while the coordinating groups in the pendant arms facilitate rapid radiolabeling kinetics.^24–29^ These hybrid chelators demonstrated high efficiency in rapidly and tightly sequestering radionuclides, including ^64^Cu, ^177^Lu, ^90^Y, and ^205/6^Bi.^23–29^ In particular, we developed promising bifunctional chelators within the NE3TA platform for ^64^Cu.^27–29^ The heptadentate NE3TA-based chelators contain seven donor atoms for coordination with Cu­(II) and are designed to effectively saturate the coordination sphere of Cu­(II).^27^ These chelators are constructed on the compact 1,4,7-triazacyclononane (TACN) backbone, which is well matched to the ionic radius of Cu­(II), thereby supporting favorable size-fit complexation.^27^ The NE3TA-based chelators exhibited excellent radiolabeling efficiency and *in vitro* stability with ^64^Cu.^27–29^ We therefore sought to apply this robust chelation platform to the development of liver-selective ^64^Cu PET tracers.

In this study, we report the development and preliminary evaluation of a liver-targeted ^64^Cu PET tracer based on a CDCA-derived NE3TA conjugate (Figure 1). The conjugate was radiolabeled with ^64^Cu by using the bifunctional chelator 3p-*C*-NE3TA at room temperature, and the corresponding ^64^Cu-labeled 3p-*C*-NE3TA-CDCA conjugate was evaluated for stability in human serum and biodistribution in healthy mice.

**Figure 1:**
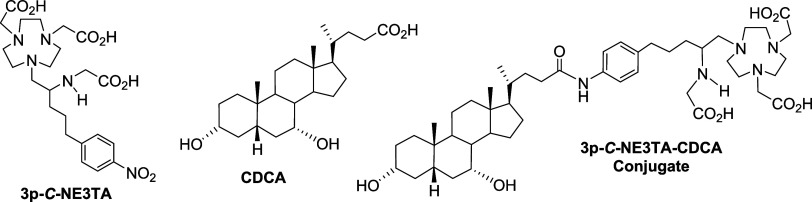
Structure of 3p-*C*-NE3TA, CDCA, and 3p-*C*-NE3TA-CDCA Conjugate.

## Results and Discussion

Synthesis of the 3p-*C*-NE3TA-CDCA conjugate (**4**) is outlined in Scheme 1. The chelator was introduced to CDCA via structural modification at the C-24 position. The nitro group in 3p-*C*-NE3TA (1)^20^ was quantitatively converted to the chelator precursor **2** in the amino form via hydrogenolysis in water at room temperature. Subsequent base-promoted coupling of **2** with the activated ester of CDCA **3** ^23^ was carried out at room temperature in a biphasic solution (THF/H_2_O) to afford the desired 3p-*C*-NE3TA-CDCA conjugate (**4**).

**Scheme 1:**
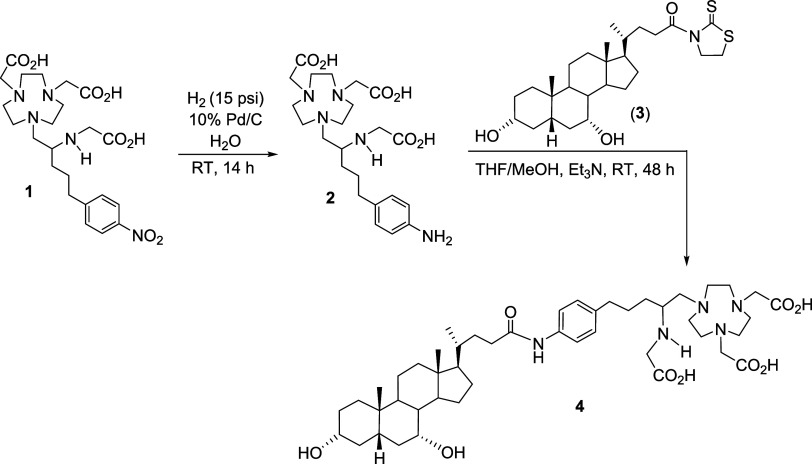
Synthesis of 3p-*C*-NE3TA-CDCA (**4**)

To assess whether conjugation of CDCA to the chelator would alter interactions of the parent bile acid with protein targets, comparative molecular docking simulations were performed using the CDCA-derived NE3TA conjugate and the parent CDCA. Crystallographic structures of CDCA-protein complexes have been reported.^31,32^ We selected the farnesoid X receptor (FXR)^33^ as a structural reference protein to evaluate the effect of conjugation on the binding conformation of the CDCA backbone. The predicted docking poses of CDCA and 3p-*C*-NE3TA-CDCA within the FXR binding pocket were compared. As shown in Figure 2, the 3p-*C*-NE3TA-CDCA conjugate adopted a binding orientation that closely overlapped with that of CDCA, indicating that attachment of the chelator did not substantially perturb the overall conformation of the CDCA framework and its binding pose in FXR.

**Figure 2:**
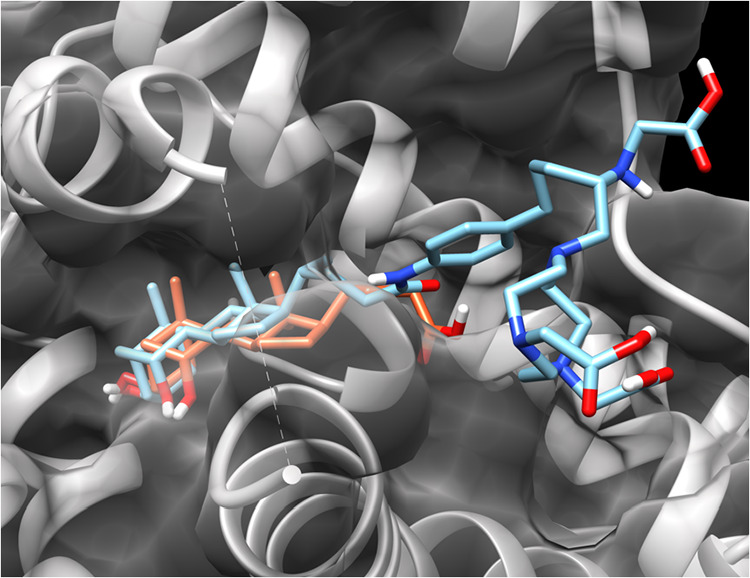
Images of CDCA (orange) and the 3p-C-NE3TA-CDCA conjugate (blue) docked in FXR (PDB: 6HL1).

The 3p-*C*-NE3TA-CDCA conjugate was evaluated for radiolabeling efficiency with ^64^Cu (Figure 3 and Supporting Information). 3p-*C*-NE3TA-CDCA in 0.25 M NH_4_OAc buffer solution (pH 7.0) was radiolabeled with ^64^Cu at room temperature. During the reaction time (1 h), the radiolabeling efficiency was monitored by TLC analysis. An aliquot of the reaction mixture was withdrawn at the designated time points and spotted onto a TLC plate eluted with the mobile phase (20 mM EDTA/0.15 M NH_4_OAc). 3p-*C*-NE3TA-CDCA rapidly sequestered ^64^Cu at room temperature, and radiolabeling was nearly complete at the starting point (>96% radiolabeling efficiency, 1 min time point). The ^64^Cu-labeled 3p-*C*-NE3TA-CDCA conjugate was freshly prepared and evaluated for *in vitro* serum stability (Figure 3 and Supporting Information). ^64^Cu-3p-*C*-NE3TA-CDCA was incubated in human serum for 2 days (37 °C, pH 7.0). Stability was assessed by measuring the radioactivity released from the ^64^Cu-labeled 3p-*C*-NE3TA-CDCA conjugate to human serum via TLC analysis. As shown by TLC chromatograms (Figure 3 and Supporting Information), ^64^Cu-3p-*C*-NE3TA-CDCA exhibited excellent stability in human serum, with a minimum radioactivity release (<0.1% loss at 48 h postincubation).

**Figure 3:**
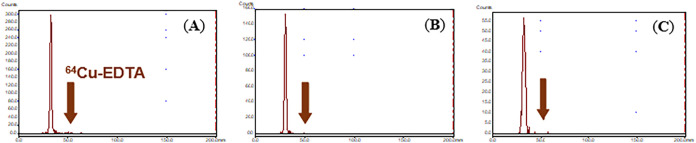
(A) TLC chromatogram of ^64^Cu-3p-*C*-NE3TA-CDCA at 1 min time point of radiolabeling with ^64^Cu at room temperature. (B) TLC chromatogram of the conjugate ^64^Cu-3p-*C*-NE3TA-CDCA in human serum at 24 h postincubation. (C) TLC chromatogram of ^64^Cu-3p-*C*-NE3TA-CDCA in human serum at 48 h postincubation. The arrow indicates ^64^Cu bound to EDTA. TLC was performed using a binary mobile phase (20 mM EDTA/0.15 M NH_4_OAc).

The *in vivo* stability and biodistribution of the ^64^Cu-labeled 3p-*C*-NE3TA-CDCA conjugate were evaluated in healthy mice (Figure 3 and Supporting Information). ^64^Cu-3p-*C*-NE3TA-CDCA was intravenously injected into female CD-1 mice (*n* = 4) via the tail vein, and radioactivity levels in blood and major organs were quantified by γ counting at 1, 4, and 24 h postinjection.

As shown in Figure 4, ^64^Cu-3p-*C*-NE3TA-CDCA exhibited low initial activity in blood (0.66% ID/g at 1 h postinjection) followed by rapid clearance from circulation (0.03% ID/g at 24 h postinjection). High hepatic uptake was observed at early time point (10.10% ID/g at 1 h), which declined substantially at 24 h postinjection (0.37% ID/g), indicating efficient hepatic clearance. Moderate uptake was observed in the lung, spleen, and kidneys at 1 h postinjection (<0.50% ID/g) and decreased at later time points. Uptake in muscle, heart, bone, and pancreas remained minimal throughout the study (<0.20% ID/g). Overall, the biodistribution profile of ^64^Cu-3p-*C*-NE3TA demonstrates rapid blood clearance, pronounced liver-selective uptake, and low accumulation in nontarget tissues.

**Figure 4:**
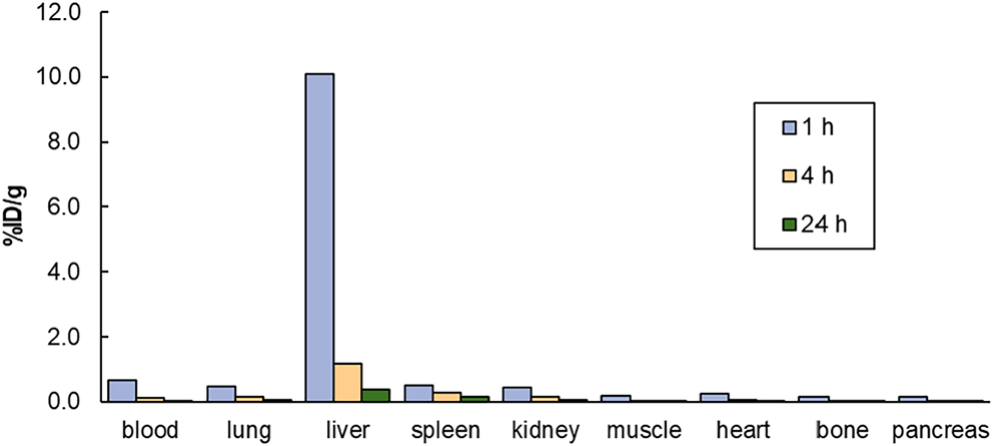
*In vivo* biodistribution of ^64^Cu-3p-*C*-NE3TA-CDCA in female CD-1 mice following intravenous injection.

## Conclusions

The highly efficient chelator 3p-*C*-NE3TA was successfully conjugated to chenodeoxycholic acid (CDCA) to generate 3p-*C*-NE3TA-CDCA as a bile acid-derived tracer platform for ^64^Cu PET imaging. The resulting ^64^Cu-labeled conjugate demonstrated efficient radiolabeling, excellent stability in human serum, and liver-selective biodistribution with favorable clearance in healthy mice. These findings support further development of ^64^Cu-3p-*C*-NE3TA-CDCA as a bile-acid-based PET tracer for imaging of liver diseases.

## Experimental Section

^1^H and ^13^C NMR spectra were obtained using a Bruker 300 NMR instrument, and chemical shifts are reported in ppm on the δ scale relative to TMS. Electrospray ionization (ESI) high-resolution mass spectra (HRMS) were obtained on a JEOL double sector JMS-AX505HA mass spectrometer (University of Notre Dame, IN). Analytical and semi-prep HPLC were performed on Agilent 1200 (Agilent, Santa Clara, CA) equipped with a diode array detector (λ = 254 and 280 nm), thermostat set at 35 °C and a Zorbax Eclipse XDB-C18 column (4.6 × 150 mm, 80 Å, Agilent, Santa Clara, CA) or a Zorbax Eclipse XDB-C18 column (9.4 × 250 mm, 80 Å, Agilent, Santa Clara, CA). The mobile phase of a binary gradient (0–100% B/15 min; solvent A, 0.1% TFA in H_2_O; solvent B, 0.1% TFA in CH_3_CN) at a flow rate of 1 mL/min was used for analytical HPLC (method 1). The mobile phase of a binary gradient (0–100% B/25 min; solvent A, 0.1% TFA in H_2_O; solvent B, 0.1% TFA in CH_3_CN at a flow rate of 3 mL/min) was used for Semi-Prep HPLC (method 2).

### 2,2′-(7-(5-(4-Aminophenyl)-2-((carboxymethyl)­amino)­pentyl)-1,4,7-triazonane-1,4-diyl)­diacetic Acid (**2**)

Compound **1** (60 mg, 0.118 mmol) was dissolved in H_2_O (8 mL). To the solution was added dry 10% Pd/C (18 mg) under argon gas. The reaction mixture was subjected to hydrogenolysis (20 psi) for 3 h at room temperature. The resulting mixture was filtered via a Celite bed and washed with ethanol. The filtrate was concentrated *in vacuo* to provide compound **2** (56 mg, 100%) as a light yellow oil in quantitative yield. Analytical HPLC (method 1, *t*_R_ = 6.3 min). ^1^H NMR (D_2_O, 300 MHz) δ 1.41–1.70 (m, 4H), 2.58–2.82 (m, 5H), 2.89–3.49 (m, 12H), 3.18–3.82 (m, 6H), 7.23 (d, *J* = 3.6 Hz, 2H), 7.30 (d, *J* = 3.9 Hz, 2H); ^13^C NMR (D_2_O, 75 MHz) δ 26.1, 27.4, 34.1, 46.0, 49.1, 49.6, 51.7, 56.6, 58.0, 58.4, 123.1, 127.8, 130.2, 143.1, 171.8. HRMS (Positive ion ESI) Calcd for C_23_H_37_N_5_O_6_ [M + H]^+^ *m*/*z* 480.2817, Found: [M + H]^+^ *m*/*z* 480.2820.

### 2,2′-(7-(2-((Carboxymethyl)­amino)-5-(4-((5-((3*S*,5*R*,7*S*,10*R*,13*S*,17*R*)-3,7-dihydroxy-10,13-dimethylhexadecahydro-1*H*-cyclopenta­[*a*]­phenanthren-17-yl)­hex-1-en-2-yl)­amino)­phenyl)­pentyl)-1,4,7-triazonane-1,4-diyl)­diacetic Acid (**4**)

Compound **2** (64 mg, 0.1 mmol) was dissolved in H_2_O (2 mL) and THF (2 mL) and treated with NaHCO_3_ (42 mg, 0.51 mmol) at room temperature. The resulting mixture was stirred and treated with Et_3_N (70 mg, 0.51 mmol) at room temperature. To the resulting mixture was added **3** (50 mg, 0.51 mmol) in THF (2 mL). The resulting mixture was stirred for 36 h. The reaction mixture was refluxed for 48 h and concentrated to dryness *in vacuo*. The residue was washed with CH_2_Cl_2_ and dissolved in EtOH, and the resulting solution was concentrated to dryness *in vacuo*. The residue was subjected to semi-prep HPLC (method 2) to isolate **4**. Analytical HPLC (method 1, *t*_R_ = 12.2 min). ^1^H NMR (MeOD, 300 MHz) δ 0.71 (s, 3H), 0.93 (s, 3H), 1.02 (d, *J* = 6.0 Hz, 3H), 1.10–2.04 (m, 29H), 2.16–2.78 (m, 7H), 2.96–3.16 (m, 9H), 3.48–3.61 (m, 2H), 3.76–4.02 (m, 6H), 7.17 (d, *J* = 8.4 Hz, 2H), 7.46 (d, *J* = 8.4 Hz, 2H). HRMS (Negative ion ESI) Calcd for C_47_H_75_N_5_O_9_ [M – H]^−^ *m*/*z* 852.5492, Found: [M – H]^−^ *m*/*z* 852.5477. ^13^C NMR data of **4** are not reported due to the insolubility of the compound in standard solvents and signal overlap with MeOD.

### Molecular Docking

Molecular docking calculations were performed to evaluate the effect of conjugation on the binding conformation of the CDCA scaffold using the farnesoid X receptor (FXR) as a structural reference protein.^32^ The optically active 3p-*C*-NE3TA-CDCA was generated by using the *S* configuration of 3p-*C*-NE3TA for docking simulations. The crystal structure of FXR (PDB ID: 6HL1)^32^ was retrieved from the Protein Data Bank. Receptor and ligand structures were prepared, and docking simulations were conducted using AutoDock Vina v1.2.0.^34^ The search space was defined by a grid box centered at coordinates (13, −13, 12) with dimensions of 20 × 30 × 20 Å. This volume encompassed the active binding pocket, which was defined based on the center of mass of the catalytic residues. Docking simulations were performed with an exhaustiveness of 32. Molecular visualization and analysis of the docking poses were performed using UCSF Chimera v1.18.3.^35^

### Radiolabeling

^64^Cu in the chloride form was purchased from Washington University (St. Louis, Missouri). The 3p-*C*-NE3TA-CDCA conjugate was evaluated for radiolabeling with ^64^Cu based on the method used for the assessment of 3p-*C*-NE3TA for comparison, as previously reported.^27^ In brief, ^64^Cu (30 μCi) was added to 3p-*C*-NE3TA-CDCA (10 μg, 10 mM) dissolved in a 0.25 M NH_4_OAc buffer solution (16 μL, pH 7.0). The reaction mixture was diluted to 20 μL using the buffer solution and agitated in a thermomixer set at 1000 rpm at room temperature for 1 h. Labeling efficiency of 3p-*C*-NE3TA-CDCA with ^64^Cu was determined by TLC (Silica gel 60 F254) using a TLC scanner (Bioscan, B-FC-100). An aliquot of the reaction mixture (2.0 μL) was withdrawn at each designated time point and spotted on the TLC plate, which was eluted with a mobile phase (20 mM EDTA/0.15 M NH_4_OAc). ^64^Cu-3p-*C*-NE3TA-CDCA and ^64^Cu bound to EDTA appeared around 30–50 and 50–70 mm, respectively.

### *In Vitro* Serum Stability

For comparison, the stability of ^64^Cu-3p-*C*-NE3TA-CDCA in human serum (GeminiBio) was determined based on the same method used for ^64^Cu-3p-*C*-NE3TA as a control.^27^ ^64^Cu-3p-*C*-NE3TA-CDCA was freshly prepared and directly used for serum stability studies without purification. Human serum (80 μL) was added to ^64^Cu-3p-*C*-NE3TA-CDCA in a microcentrifuge tube. The stability of ^64^Cu-3p-*C*-NE3TA-CDCA incubated in human serum at 37 °C for 2 days was assessed by using TLC (eluent: 20 mM EDTA/0.15 M NH_4_OAc). The complexes ^64^Cu-3p-*C*-NE3TA-CDCA and ^64^Cu bound to EDTA appeared around 30–50 and 50–70 mm, respectively.

### *In Vivo* Biodistribution of Study

The animal study was performed in compliance with the Guidelines for Care and Use of Research Animals established by Washington University’s Animal Studies Committee. The biodistribution study was carried out in female CD-1 mice (Charles River Laboratories). ^64^Cu-NE3TA-CDCA was freshly prepared and directly used for *in vivo* studies without further purification. The tissue uptakes of ^64^Cu-3p-*C*-NE3TA-CDCA were evaluated in mice (*n* = 4) that were injected via the tail vein with ^64^Cu-3p-*C*-NE3TA-CDCA (0.51 MBq, 19 μCi) in saline (100 μL) per animal. At 1, 4, and 24 h postinjection, mice were anesthetized with 1–2% isoflurane and sacrificed by cervical dislocation. Subsequently, the tissues of interest were harvested, weighed, and measured on a γ counter. Samples were corrected for radioactive decay to calculate the percent injected dose per gram (%ID/g) of tissue.

## Supplementary Material



## Notes

Copies of NMR spectra and copies of TLC chromatograms for assessment of radiolabeling reaction kinetics and serum stability, and *in vivo* biodistribution data (PDF)
