# Supplementary material for: CDCA-Derived NE3TA Conjugate for Liver-Selective 64Cu PET Imaging
Source: ACS Omega. 2026 Apr 1;11(14):22228–33. doi: 10.1021/acsomega.5c13607 (PMC13084480; doi:10.1021/acsomega.5c13607)
Supplement: Supplementary file 1 [file ao5c13607_si_001.pdf]

Supporting Information

CDCA-Derived NE3TA Conjugate for Liver Selective ^{64}Cu PET imaging

Haixing Wang,[1] Siyuan Ren,[1] Nilantha Bandara,[2] Shuyuan Zhang,[1] Rachael Fujimori,[1]
Hong Ha Nguyen,[1] David D. L. Minh,[1] Buck E. Rogers,[2] Hyun-Soon Chong[1,*]

[1]Department of Chemistry, Illinois Institute of Technology, Chicago, IL, USA;
[2]Department of Radiation Oncology, Washington University, Saint Louis, MO, USA.

Table of Contents

I. NMR Spectra of Compounds **2** and **4** -- S2-S3

II. HPLC chromatograms of Compounds **3** and **4** -- S3 -S4

III. Radiolabeling efficiency of 3p-*C*-NE3TA-CDCA with ^{64}Cu (TLC chromatograms) --- S4-S5

IV. In vitro serum stability of ^{64}Cu-3p-*C*-NE3TA-CDCA (TLC chromatograms) ----------- S6

V. *In vivo* biodistribution data of ^{64}Cu-3p-*C*-NE3TA-CDCA ---------------------------------- S7

I. NMR Spectra of Compounds **2** and **4**

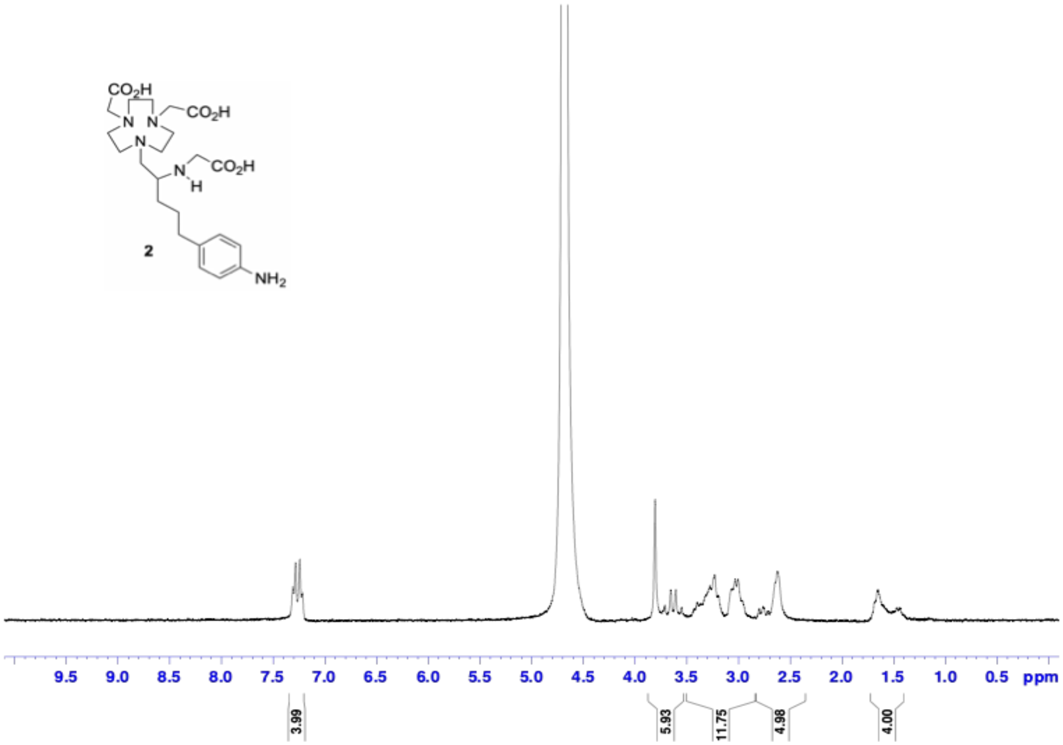

Figure S1. ¹H NMR spectrum of Compound 2

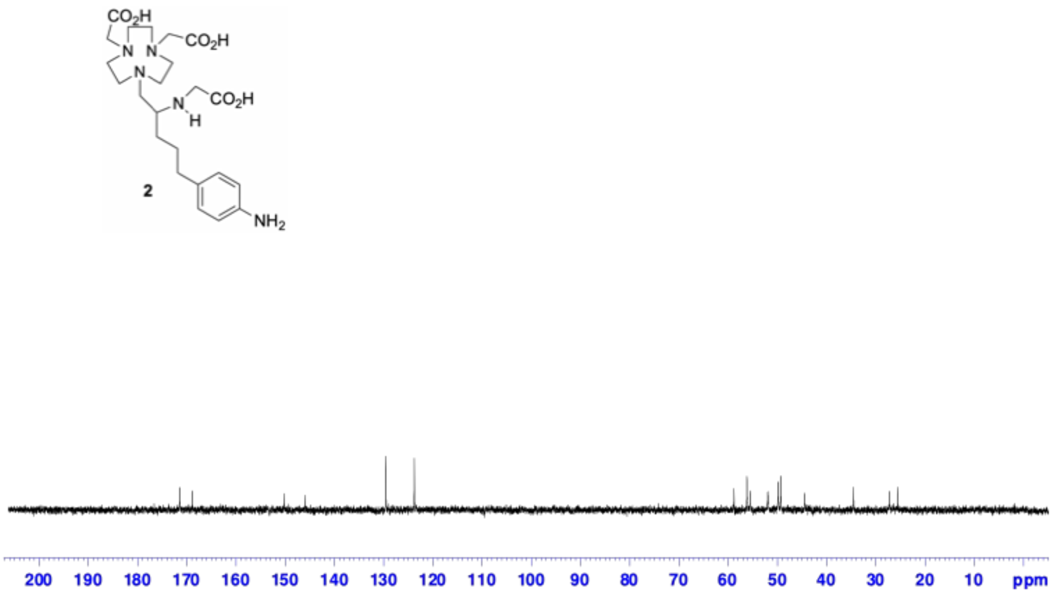

Figure S2. ¹³C NMR spectrum of Compound 2

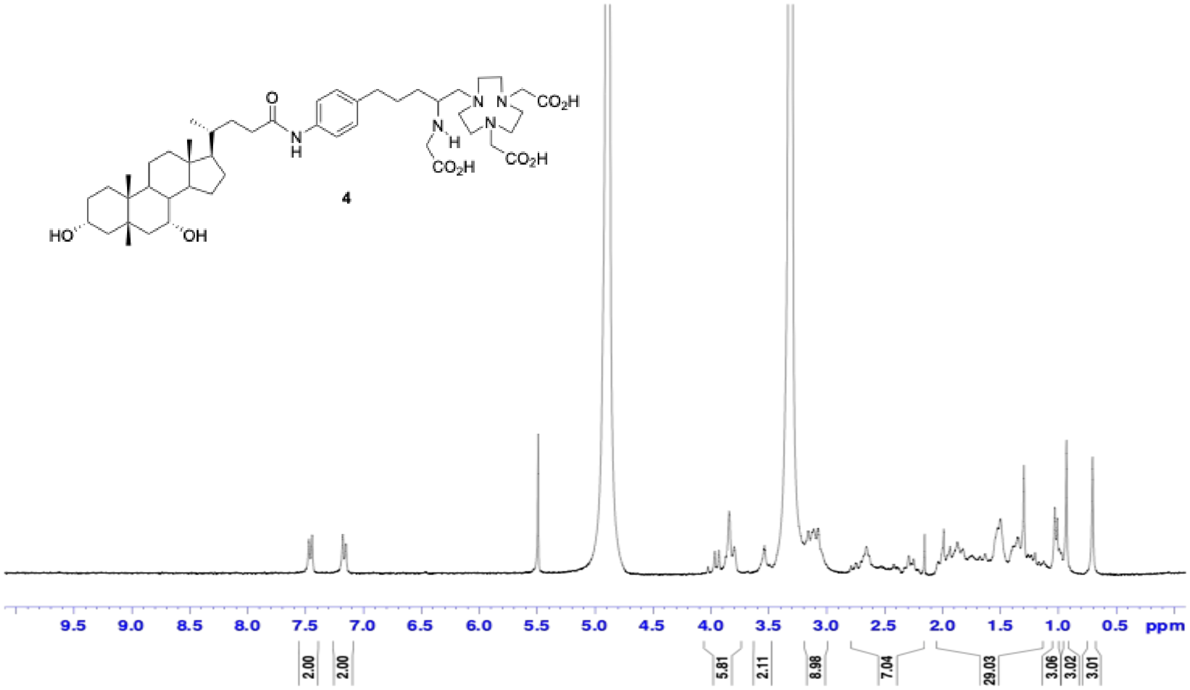

Figure S3. ^{1}**H NMR spectrum of Compound 4**

II. HPLC chromatograms of compounds 2 and 4.

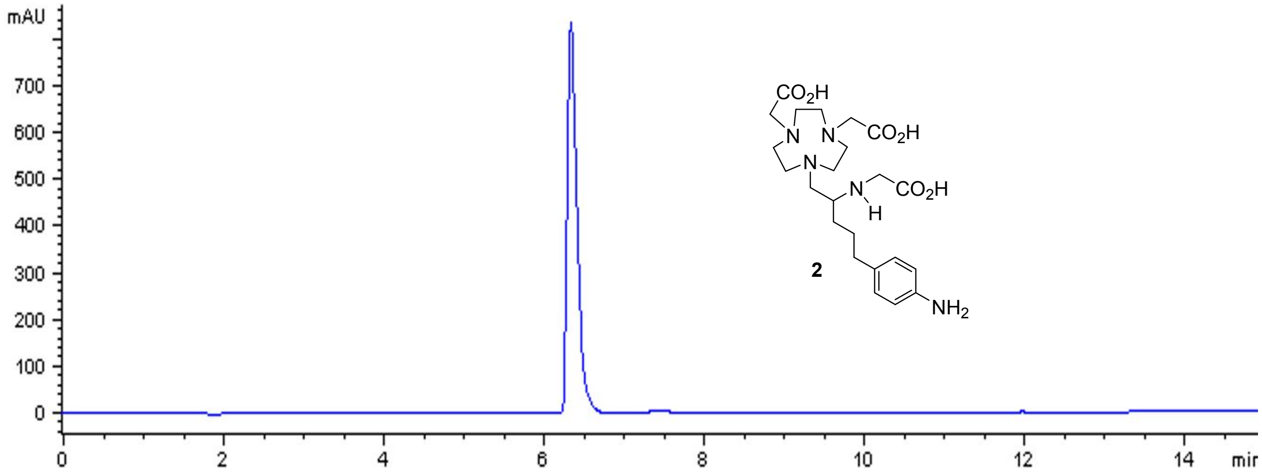

Figure S4. HPLC chromatogram of Compound 2

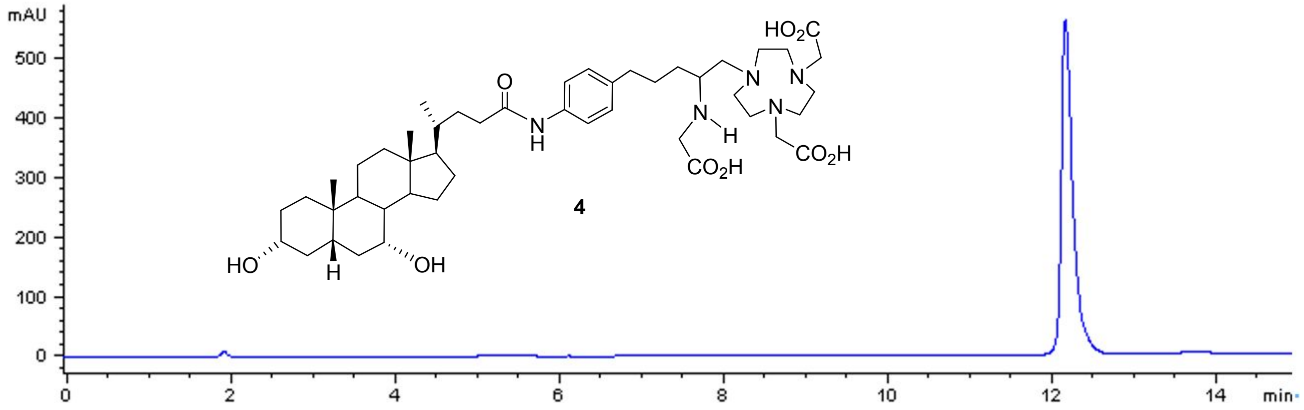

Figure S5. HPLC chromatogram of Compound 4

III. Radiolabeling efficiency of 3p-*C*-NE3TA-CDCA with ^{64}Cu (pH 7.0, room temperature).

Table S1. Radiolabeling efficiency (mean ± standard deviation%) measured in duplicate using TLC and a binary eluent (20 mM EDTA/0.15 M NH_4OAc).

Time	Radiolabeling efficiency (%)
1 min	99.7 ± 0.1
10 min	99.7 ± 0.2
30 min	99.7 ± 0.0
60 min	99.8 ± 0.1

Figure S6. Control TLC chromatogram of unbound radionuclide ($^{64}CuCl_2$)

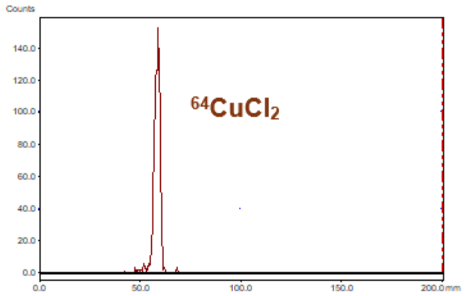

Figure S7. TLC chromatograms for radiolabeling of 3p-C-NE3TA-CDCA with ^{64}Cu.

1st run

1 min
99.6%

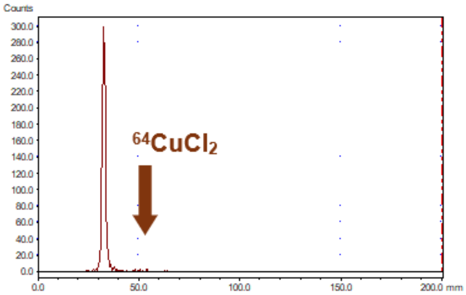

10 min
99.8%

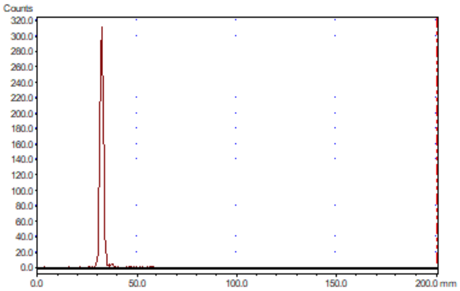

30 min
99.7%

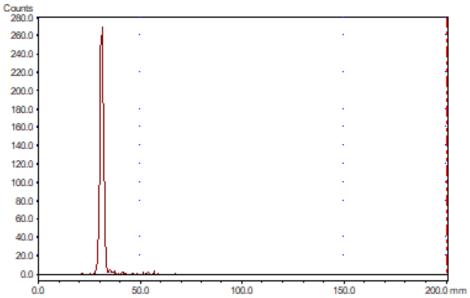

60 min
99.7%

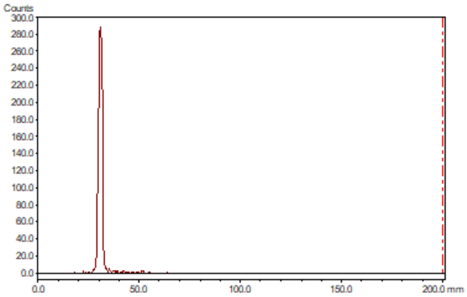

2st run

1 min
99.7%

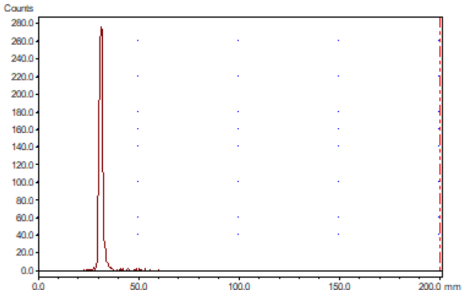

10 min
99.5%

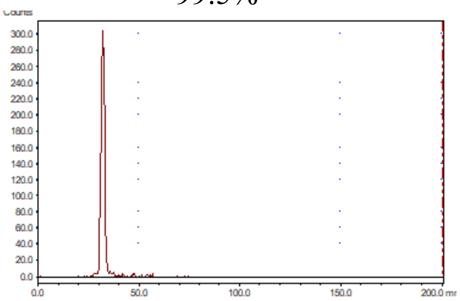

30 min
99.7%

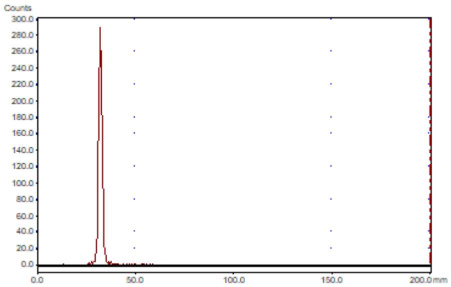

60 min
99.9%

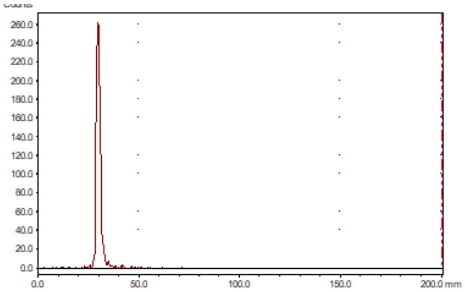

IV. In vitro serum stability of ^{64}Cu-3p-*C*-NE3TA-CDCA (37 ºC and pH 7.4)

Table 2. In vitro serum stability (mean ± standard deviation%) measured in duplicate using TLC and a binary eluent (20 mM EDTA/0.15 M NH_4OAc).

Time	^{64}Cu-3p-*C*-NE3TA-CDCA (%)
0 h	99.7 ± 0.14
24 h	99.9 ± 0.14
48 h	99.95 ± 0.07

Figure S8. TLC chromatograms for stability of ^{64}Cu-3p-*C*-NE3TA-CDCA in human serum.

0 hr

Sample 1
99.8%

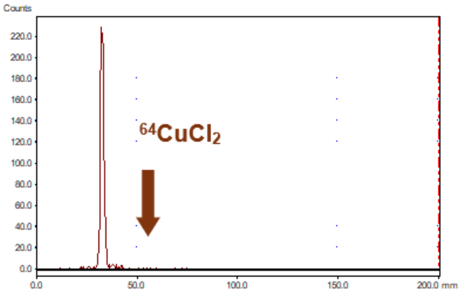

Sample 2
99.6%

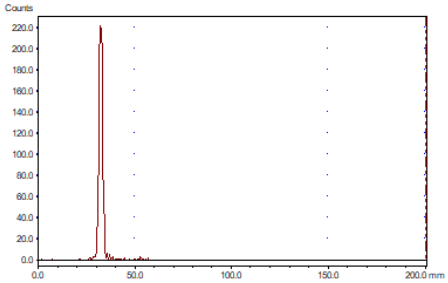

24 hr

Sample 1
100.0%

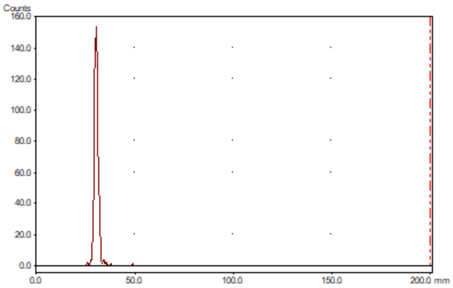

Sample 2
99.8%

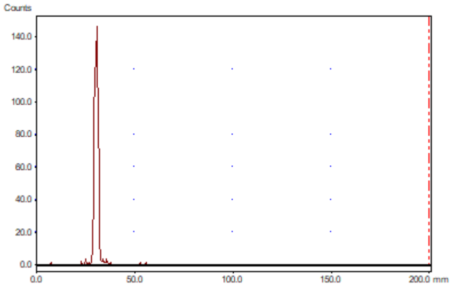

48 hr

Sample 1
100.0%

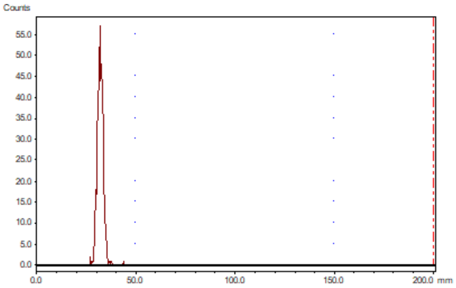

Sample 2
99.9%

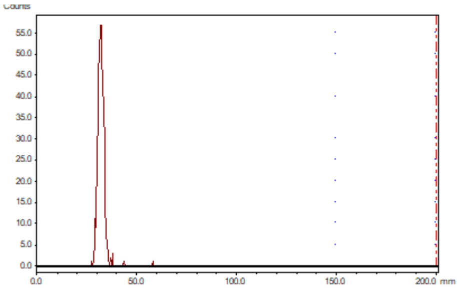

V. *In vivo* biodistribution of ^{64}Cu-*C*-NE3TA-CDCA in healthy mice.

Table 3. *In vivo* biodistribution data of ^{64}Cu-*C*-NE3TA-CDCA in female CD-1 mice following intravenous injection (n = 4).

Tissue	Time points (mean ± SD, %ID/g)		
	1 h	4 h	24 h
blood	0.6607 ± 0.0880	0.1345 ± 0.0076	0.0270 ± 0.0076
lung	0.4908 ± 0.0560	0.1448 ± 0.0194	0.0667 ± 0.0165
liver	10.1041 ± 1.8106	1.1619 ± 0.1041	0.3714 ± 0.0437
spleen	0.4974 ± 0.1412	0.2991 ± 0.0385	0.1549 ± 0.0338
kidney	0.4540 ± 0.1102	0.1437 ± 0.0067	0.0710 ± 0.0228
muscle	0.1906 ± 0.0362	0.0265 ± 0.0076	0.0063 ± 0.0042
heart	0.2559 ± 0.0314	0.0568 ± 0.0054	0.0385 ± 0.0126
bone	0.1474 ± 0.0745	0.0386 ± 0.0196	0.0287 ± 0.0334
pancreas	0.1672 ± 0.0956	0.0332 ± 0.0082	0.0210 ± 0.0034
